# Inhibition of SypG-Induced Biofilms and Host Colonization by the Negative Regulator SypE in *Vibrio fischeri*

**DOI:** 10.1371/journal.pone.0060076

**Published:** 2013-03-28

**Authors:** Andrew R. Morris, Karen L. Visick

**Affiliations:** Department of Microbiology and Immunology, Loyola University Medical Center, Maywood, Illinois, United States of America; Institut Pasteur, URA CNRS 2172, France

## Abstract

*Vibrio fischeri* produces a specific biofilm to promote colonization of its eukaryotic host, the squid *Euprymna scolopes*. Formation of this biofilm is induced by the sensor kinase RscS, which functions upstream of the response regulator SypG to regulate transcription of the symbiosis polysaccharide (*syp*) locus. Biofilm formation is also controlled by SypE, a multi-domain response regulator that consists of a central regulatory receiver (REC) domain flanked by an N-terminal serine kinase domain and a C-terminal serine phosphatase domain. SypE permits biofilm formation under *rscS* overexpression conditions, but inhibits biofilms induced by overexpression of *sypG*. We previously investigated the function of SypE in controlling biofilm formation induced by RscS. Here, we examined the molecular mechanism by which SypE naturally inhibits SypG-induced biofilms. We found that SypE’s N-terminal kinase domain was both required and sufficient to inhibit SypG-induced biofilms. This effect did not occur at the level of *syp* transcription. Instead, under *sypG*-overexpressing conditions, SypE inhibited biofilms by promoting the phosphorylation of another *syp* regulator, SypA, a putative anti-sigma factor antagonist. Inhibition by SypE of SypG-induced biofilm formation could be overcome by the expression of a non-phosphorylatable SypA mutant, indicating that SypE functions primarily if not exclusively to control SypA activity via phosphorylation. Finally, the presence of SypE was detrimental to colonization under *sypG*-overexpressing conditions, as cells deleted for *sypE* outcompeted wild-type cells for colonization when both strains overexpressed *sypG*. These results provide further evidence that biofilm formation is critical to symbiotic colonization, and support a model in which SypE naturally functions to restrict biofilm formation, and thus host colonization, to the appropriate environmental conditions.

## Introduction

Bacterial biofilms, or surface-associated communities of cells encapsulated in an extracellular matrix, are ubiquitous in the environment and likely represent the preferred lifestyle mode for many bacterial species [1]. The formation of a biofilm can impact multiple aspects of a bacterium’s physiology, including sensitivity to antibiotics and other antimicrobials [2], cellular metabolism [3], and gene expression [4]. Biofilms have also been demonstrated to play critical roles in mediating interactions between bacteria and their eukaryotic hosts [5], [6].

**Figure 1:**
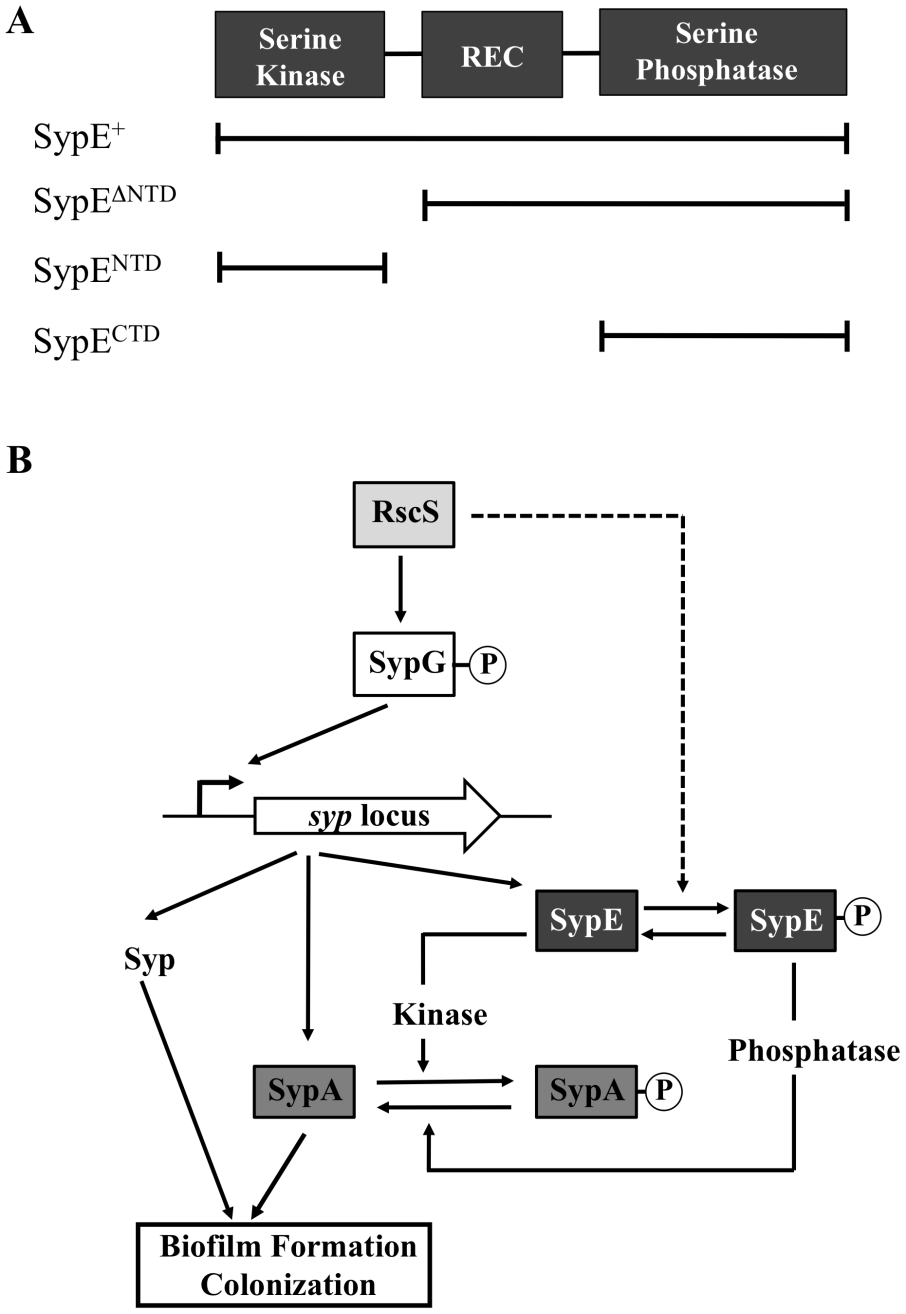
Model of inhibition of SypG-induced biofilms by SypE. (A) Schematic of the domain structure of SypE and select SypE mutants. SypE contains a central regulatory receiver (REC) domain flanked by a N-terminal HPK-like serine kinase domain and a C-terminal PP2C-like serine phosphatase domain. The black lines represent select SypE mutants containing the indicated protein domains. (B) When *sypG* is overexpressed, transcription of the *syp* locus is activated, resulting in the production of Syp structural proteins necessary for polysaccharide production and biofilm formation, as well as the production of regulatory proteins SypA and SypE. Our data here show that, under these conditions, SypE functions as a serine kinase and phosphorylates the downstream target protein, the putative anti-sigma factor antagonist protein SypA. Phosphorylated SypA is inactive to promote biofilm formation and host colonization. In contrast, when *rscS* is overexpressed, the kinase activity of SypE is inactivated (presumably through phosphorylation of SypE’s REC domain); instead, it functions as a serine phosphatase to dephosphorylate SypA, which promotes biofilm formation and colonization through an unknown mechanism. Co-overexpression of *sypG* and *sypA* (not shown) leads to biofilm formation, presumably because high levels of SypA permit some SypA to escape phosphorylation and inactivation by SypE.

The symbiotic association between the marine bacterium *Vibrio fischeri* and its host the Hawaiian squid *Euprymna scolopes* provides a natural model to study the bacterial processes necessary to promote host colonization [7], [8], [9]. Importantly, one of the earliest stages of the colonization process involves the formation of a specific biofilm or bacterial aggregate on the surface of the squid’s symbiotic light organ [10]. Formation of this biofilm requires the symbiosis polysaccharide (*syp*) locus, which is regulated at the transcriptional level by the sensor kinase (SK) RscS and the *syp*-encoded response regulator (RR) SypG [5], [11], [12]. As a result, both *rscS* and *sypG* are required for initiation of host colonization [12], [13]. The current model predicts that, upon detection of an as-yet unidentified signal(s), RscS autophosphorylates and serves as a phosphodonor to ultimately activate the RR SypG [12] (Fig. 1). Phosphorylated SypG is thought to directly promote transcription of the individual *syp* operons, which encode the structural genes necessary for polysaccharide production and thus biofilm formation [5], [14].

The natural signal(s) that activate RscS remain unknown, and thus conditions under which wild-type cells form *syp*-dependent biofilms, other than during symbiosis, have not been established. However, overexpression of either *rscS* or *sypG* can promote the production of biofilms in laboratory culture, and provides a powerful tool to dissect the regulatory components involved in this signaling pathway. Overexpression constructs have been similarly employed in a variety of bacterial systems in which the natural signals remain unidentified, such as *Vibrio cholerae* and *Salmonella enterica* serovar Typhimurium [15], [16], [17]. The overexpression of *rscS* in wild-type cells results in the activation of *syp* transcription and *syp*-dependent biofilm formation, in particular the formation of wrinkled colonies on solid agar media and pellicles in static liquid cultures [11], [12]. The formation of these biofilms depends on an intact, functional SypG [12]. *rscS* overexpression also results in increased symbiotic aggregation, which correlates with a colonization advantage over wild-type cells [11].

Intriguingly, overexpression of *sypG* in wild-type cells similarly induces activation of the *syp* locus, but fails to promote biofilm phenotypes as observed for RscS [12]. In a previous study, Hussa *et al*. (2008) demonstrated that the inability of *sypG* overexpression to induce *syp* biofilm formation was due to the inhibitory activity of a second *syp-*encoded RR, SypE; overexpression of *sypG* in a Δ*sypE* mutant resulted in dramatic biofilm formation similar to that observed with *rscS*-overexpressing wild-type cells [12]. Complementation with a wild-type allele of *sypE* fully restored the wild type, non-biofilm-forming phenotypes [12]. Two main conclusions can be drawn from these studies: 1) RscS and SypG both function to induce biofilm formation, and 2) RscS may have an additional function in controlling SypE activity that SypG lacks.

SypE is a multi-domain RR consisting of a central RR receiver (REC) domain flanked by an N-terminal, serine kinase domain and a C-terminal, serine phosphatase domain (Fig. 1A) [18], [19], [20]. The mechanism by which SypE naturally inhibits biofilms under SypG-inducing conditions has not yet been investigated. However, we recently characterized the role of SypE in biofilms formed when *rscS* is overexpressed. Under these conditions, deletion of *sypE* slightly, but reproducibly delayed the formation of wrinkled colonies. This phenotype could be complemented by expression of the full-length SypE protein from an exogenous location in the chromosome. In addition, it could be complemented by expression of SypE’s C-terminal, serine phosphatase domain alone. Surprisingly, however, expression of SypE’s N-terminal kinase domain alone not only failed to complement, but in fact fully inhibited biofilms induced by *rscS* overexpression [19]. These data support the idea that RscS must modulate the activity of SypE such that it functions primarily to promote biofilm formation.

These studies also predicted that SypE regulates biofilms by controlling the phosphorylation state of a downstream regulatory protein. That downstream regulator has since been identified as SypA, a putative anti-sigma factor antagonist (Fig. 1B) [20]. Under *rscS*-overexpressing conditions, SypA is largely unphosphorylated and promotes biofilm formation [20]. Furthermore, SypA is largely phosphorylated, even when *rscS* is overexpressed, when the cells express a mutant SypE protein (SypE^D192A^) that constitutively inhibits biofilm formation. SypE thus mediates control over biofilms induced by *rscS* overexpression by controlling the phosphorylation state of SypA [20].

These previous studies investigated the regulation of biofilms induced by overexpression of *rscS*, a condition in which SypE functions as a positive regulator. Our goal here was to further our understanding of SypE function by determining whether its ability to naturally inhibit biofilm formation induced by *sypG* overexpression occurs through mechanisms similar to that observed with RscS overexpression. Indeed, we obtained compelling evidence that under *sypG* overexpressing conditions, SypE functions as a kinase to inhibit biofilm formation: (1) SypE’s N-terminal kinase domain was both required and sufficient to mediate inhibition of SypG-induced biofilms and (2) SypE inhibits biofilms in *sypG*-overexpressing cells by promoting phosphorylation, and thus inactivation, of the regulatory protein SypA. In addition, we further extend our understanding of SypE function by determining that the impact of SypE occurs at a level downstream of *syp* transcription. Finally, we find that the ability of SypE to inhibit biofilm formation in laboratory culture is mirrored in its ability to inhibit symbiotic colonization of the squid host. Together, these data demonstrate that the negative regulation of SypG-induced biofilms by SypE is a critical mechanism by which *V. fischeri* restricts host colonization.

## Materials and Methods

### Bacterial Strains, Plasmids, and Media

Wild-type (WT) *V. fischeri* ES114 [21] was used as the parent strain for these studies. Strains derived from ES114 and plasmids utilized in this study are listed in Tables 1 and 2, respectively. *E. coli* strains TAM1 (Active Motif, Carlsbad, CA), TOP10 F (Invitrogen, Carlsbad, CA), and GT115 (InvivoGen, San Diego, CA) were used for cloning. To generate Tn*7* insertions in *V. fischeri,* tetraparental matings were carried out as previously described [22]. To generate the *sypG* overexpression plasmid pCLD56, restriction digest was utilized to remove the Cm^R^ cassette from plasmid pEAH73 [12]. To generate the various *sypE* and *sypA* alleles, we performed PCR using the primers listed in Table 3. The resulting PCR products were cloned into the cloning vector pJET1.2 (Fermentas, Glen Burnie, MD) and then subcloned into mobilizable plasmid pVSV105 [23], pKV282 [19], or pEVS107 [22]. *V. fischeri* strains were grown in LBS [24] or sea water tryptone (SWT) [5]. *Escherichia coli* strains were grown in LB [25] or brain heart infusion medium (Difco, Detroit, MI). The following antibiotics were added to *V. fischeri* media, where necessary, at the indicated concentrations: chloramphenicol (Cm) 2.5 µg mL^−1^, erythromycin (Em) at 5 µg mL^−1^, and tetracycline (Tc) at 5 µg mL^−1^ in LBS and 30 µg mL^−1^ in SWT. The following antibiotics were added to *E. coli* media, where necessary, at the indicated concentrations: Cm at 25 µg mL^−1^, kanamycin (Kan) at 50 µg mL^−1^, Tc at 15 µg mL^−1^, or ampicillin (Ap) at 100 µg mL^−1^. For solid media, agar was added to a final concentration of 1.5%.

**Table 1:** *V. fischeri* strains used in this study.

Strain	Relevant Genotype	Source or Reference
ES114	Wild-type V. *fischeri*	[21]
KV3246	*att*Tn*7::*P*sypA*-*lacZ* em^R^	This study
KV3299	Δ*sypE*	[12].
KV4389	*att*Tn*7*:: em^R^	[19]
KV4390	Δ*sypE att*Tn*7*:: em^R^	[19]
KV4715	Δ*sypA*	[20]
KV4716	Δ*sypA* Δ*sypE*	[20]
KV4819	Δ*sypE att*Tn*7*::*sypE* em^R^	[19]
KV4926	Δ*sypE att*Tn*7*::P*sypA*-*lacZ* em^R^	This study
KV5479	Δ*sypA att*Tn*7*:: *sypA* ^+^ em^ R^	[20]
KV5481	Δ*sypA att*Tn*7*:: *sypA* ^S56A^ em^R^	[20]
KV5649	Δ*sypA* Δ*sypE attn*Tn*7*:: *sypE* em^R^	[20]
KV6578	Δ*sypA att*Tn*7*:: *sypA*-HA em^R^	This study
KV6579	Δ*sypA att*Tn*7*:: *sypA* ^S56A^-HA em^R^	This study
KV6580	Δ*sypA* Δ*sypE attn*Tn*7*:: *sypA*-HA em^R^	This study

**Table 2:** Plasmids used in this study.

Name	Description	Relevant primers^1^	Source or Reference
pARM3	pVSV105+1.2 kb SypE^ΔNTD^; cm^R^	256, 868	This study
pARM4	pVSV105+ *sypE* ^N52A^; cm^R^	849, 876	This study
pARM7	RscS overexpression construct; tet^R^	N/A	[19]
pARM9	pVSV105+1.6 Kb *sypG;* cm^R^	393, 559	This study
pARM13	pKV282+770 bp *sypA;* tet^R^	N/A	[20]
pARM35	pKV282+770 bp *sypA*-FLAG; tet^R^	N/A	[20]
pARM111	pVSV105+700 bp *sypE* ^CTD^-FLAG; cm^R^	910, 921	This Study
pARM162	pVSV105+1.2 kb SypE^ΔNTD^-FLAG; cm^R^	868, 921	[20]
pARM163	pEVS107+1.1 kb *sypA-*HA*;* kan^R^, erm^R^	1040, 806	This study
pARM164	pEVS107+1.1 kb *sypA* ^S56A^ *-*HA*;* kan^R^, em^R^	1040, 806	This study
pCLD48	pVSV105+1.5 kb *sypE*; cm^R^	N/A	[12]
pCLD56	pKV282+1.6 Kb *sypG*; tet^R^	393, 559	This study
pCLD64	pVSV105+500 bp *sypE* ^NTD^; cm^R^	461, 911	This study
pCLD65	pVSV105+500 bp *sypE* ^NTD, N52A^; cm^R^	461, 911	This study
pCLD67	pVSV105+700 bp *sypE* ^CTD^; cm^R^	256, 910	This study
pEAH90	pEVS107+ P_sypA_-*lacZ*	N/A	[26]
pEVS104	Conjugal helper plasmid (*tra trb*), kan^R^	N/A	[36]
pEVS107	Mini-Tn*7* delivery plasmid; mob; kan^R^, em^R^	N/A	[22]
pKV282	Mobilizable vector; tet^R^	N/A	[19]
pVSV105	Mobilizable vector; cm^R^	N/A	[23]

1 Relevant primers for constructs generated in this study.

**Table 3:** Primers used in this study.

Primer name	Sequence^1^
256	tttttctgcacTTATTGATTCTCAATTAACAGC
393	GCTACACTTTCACTAGACGC
461	CATATGGCACGATGGGATCC
559	ggtaccTCATTCCGATTCTTCATAG
806	AGCTTCTTCCTTATAGTTATGATG
849	CCTGTGTGAAATTGTTATCCG
868	GTGGTGTAATCATGGAGCGTTCCCCTTCCCAT
876	TCTGAATGGAGCACCgcTCTAGTTTTGCACCCT
910	GTGGTGTAATCATGGCCCATACTCTATTACCACAA
911	CTTAATGGGAAGGGGAACGCTC
921	acccgggttatttatcatcatcatctttataATCTTGATTCTCAATTAACAG
1040	acccgggttatgcataatctggaacatcatatggataATGCGTTGTTTTATTAACAGG

1 Non-native sequences are shown in lower case letters.

### Wrinkled Colony Assays

To observe wrinkled colony formation, the indicated *V. fischeri* strains were streaked onto LBS agar plates containing Tc and Cm overnight. Single colonies were subsequently cultured with shaking in LBS broth with Tc at 28°C overnight and then sub-cultured to an optical density at 600 nm (OD_600_) of 0.2 in 5 mL of fresh medium. Cells were spun down, washed twice in 70% artificial seawater (ASW), and re-suspended in 70% ASW and diluted to an OD of 0.2. 10 µL of re-suspended cultures were spotted onto LBS agar plates and grown overnight at 28°C [26]. Images of spotted cultures were acquired at the indicated time points using the Zeiss Stemi 2000-C dissecting microscope as previously described [26].

### Static Pellicle Assays

Strains were grown with shaking in LBS containing Tc and Cm at 28°C overnight and then subcultured to an OD of 0.1 in 1.5 mL of fresh medium in 24-well microtiter dishes. Cultures were then incubated at 28°C for 48 h. The strength of each pellicle was evaluated by disrupting the air-liquid interface with a sterile pipette tip after 48 h of incubation. Cultures with little/no pellicle were scored as (–); cultures with a strong pellicle that remained intact following disruption were scored as (+). Images of pellicles were acquired at the indicated time points using a Zeiss Stemi 2000-C dissecting microscope.

### SypE FLAG Protein Expression

FLAG epitope fusions were generated to the C-terminus of the *sypE* ΔNTD and CTD mutants using the reverse primer 921 and the forward primers 868 and 910, respectively (Table 3). The FLAG-*sypE* alleles were cloned into the expression vector pVSV105 and subsequently conjugated into Δ*sypE* cells containing the *sypG* expressing plasmid (pCLD56). Strains were cultured in LBS containing Tc and Cm for 18 h at 28°C. 1 mL of overnight culture was pelleted by centrifugation, lysed in 500 mL 2X SDS sample buffer (4% SDS, 40 mM Tris pH 6.3, 10% glycerol), and resolved on 15% SDS-PAGE gels. Protein transfer to PVDF membrane was performed using standard Tris-Glycine transfer buffer (20% MeOH, 50 mM Tris, 40 mM glycine). Western blotting was performed using standard protocols with rabbit anti-FLAG primary antibody (Sigma-Aldrich, St. Louis, MO) and donkey anti-rabbit secondary antibody conjugated to horseradish peroxidase (Sigma-Aldrich, St. Louis, MO). Blots were imaged by chemiluminescent detection (SuperSignal West Pico Chemiluminescent Substrate, Pierce Biotechnology, Inc., Rockford, IL.).

### Analysis of SypA Phosphorylation Using Phos-tag™ Acrylamide

Hemagglutinin (HA) and FLAG-epitope fusions to the C-terminus of SypA were generated using the primers listed in Table 3. The *sypA-*HA alleles were cloned into the Tn*7* delivery plasmid pEVS107 [22] (Table 2). The resulting plasmids were used to introduce the *sypA-*HA alleles at the Tn*7* site of the Δ*sypA* and Δ*sypA* Δ*sypE* strains. Subsequently, the *sypG* overexpression plasmid pCLD56 was introduced. The resulting *V. fischeri* strains were streaked onto LBS agar plates containing Tc and single colonies were then cultured overnight in LBS containing Tc at 28°C with shaking. Aliquots of cells (1 mL) were spun down, washed twice with 1X PBS, and standardized to the same amounts using OD_600_ measurements. Samples were lysed in 2X SDS sample buffer and resolved on SDS-PAGE gels containing 30 µM Phos-tag™ acrylamide (WAKO chemicals, Richmond, VA) and 50 µM MnCl_2_. We observed some variation in the extent to which phospho-SypA migration was retarded relative to SypA, perhaps due to aging/integrity of the Phos-tag™ acrylamide. Gels were fixed for 15 min in standard transfer buffer containing 1 mM EDTA to remove Mn^2+^ from the gel. Gels were incubated for an additional 20 min in transfer buffer without EDTA. Proteins were transferred to a PVDF membrane and the proteins were detected by western blot analysis using an anti-HA antibody (Sigma-Aldrich, St. Louis, MO).

Similar approaches were used to assess SypA phosphorylation under overexpressing conditions with the exceptions that (1) two antibiotics were added (Cm and Tc) throughout to maintain the two (*sypG*- and *sypA*-overexpressing) plasmids and (2) the *sypA* alleles were FLAG-tagged and thus detected using an anti-FLAG antibody (Sigma-Aldrich, St. Louis, MO).

### β-galactosidase Assays

The indicated strains were grown (in triplicate) with shaking in LBS containing Tc and Cm at 28°C overnight and then sub-cultured into fresh medium and grown for up to 24 h. Aliquots (1 mL) of cells were removed at 8 h and 24 h post-inoculation, concentrated, resuspended in Z-buffer, and lysed. The β-galactosidase activity [27] and total protein concentration [28] of each sample were assayed. β-galactosidase units are reported as units of activity per mg of protein.

### Squid Colonization Assays

Experiments involving *E. scolopes* animals were carried out using approaches described in an Animal Component of Research Protocol (ACORP) approved by Loyola University’s Institutional Animal Care and Use Committee (IACUC) (LU #107314, 201297). To perform competitive colonization assays, juvenile squid were placed into artificial seawater (Instant Ocean; Aquarium Systems, Mentor, OH) containing approximately 1,000 *V. fischeri* cells per mL of seawater. Juvenile squid were inoculated with an approximate 1∶1 ratio of mutant and wild-type cells, and colonization was allowed to proceed for 18 h. For these assays, Δ*sypE* cells were marked with an erythromycin resistance (Em^R^) cassette within the chromosome at the Tn*7* site. Reciprocal experiments were also performed in which wild-type cells contained the Em^R^ marker. The ratio of bacterial strains within the light organs of the animals was assessed through luminescence and homogenization/plating assays as described previously [11]. The competitive colonization data are reported as the Log-transformed Relative Competitive Index (Log RCI).

## Results

### SypE’s N-terminal Domain is Necessary and Sufficient to Inhibit SypG Biofilms

**Figure 2:**
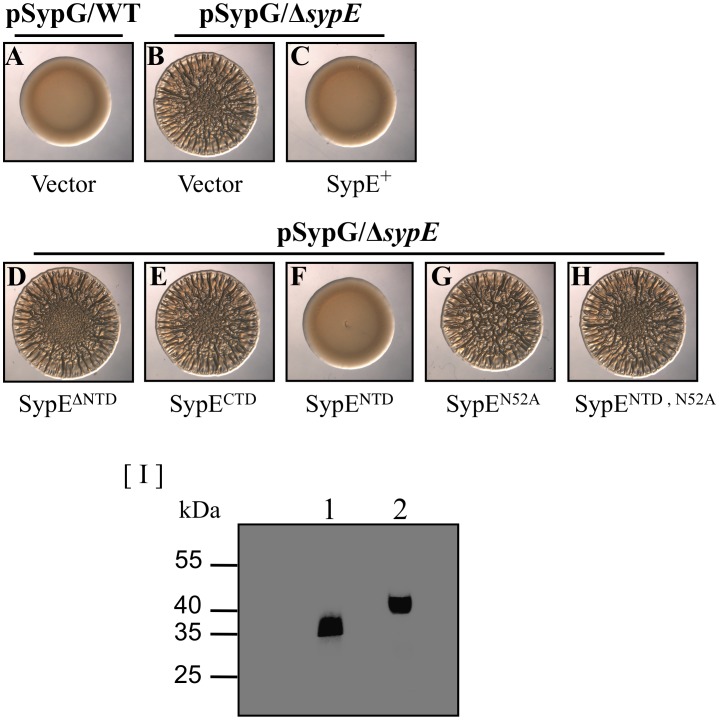
Regulation of SypG-induced wrinkled colony formation by SypE. The SypG expression plasmid (pCLD56) was introduced into either wild-type cells [A] or Δ*sypE* mutant cells [KV3299] carrying either empty vector (pVSV105) [B] or the indicated SypE-complementing plasmids: full-length SypE (pCLD48)[C], SypE^ΔNTD^ (pARM3)[D], SypE^CTD^ (pCLD67)[E], SypE^NTD^ (pCLD64)[F], SypE^N52A^ (pARM4)[G], SypE^NTD, N52A^ (pCLD65)[H]. Cultures were spotted onto agar plates and wrinkled colony morphology was assessed at 48 h post-spotting. Images are representative of at least three independent experiments. [I] Expression of FLAG epitope-tagged SypE mutant proteins was assessed by western blot analysis. Plasmids expressing FLAG-*sypE* ^CTD^ (pARM111; lane 1) or *sypE* ^ΔNTD^ (pARM162; lane 2) alleles were introduced into the Δ*sypE* strain containing the *sypG* overexpression plasmid (pCLD56). Whole-cell lysates were resolved using SDS-PAGE and the FLAG-tagged proteins were detected by western blot analysis as described in the Materials and Methods.

**Figure 3:**
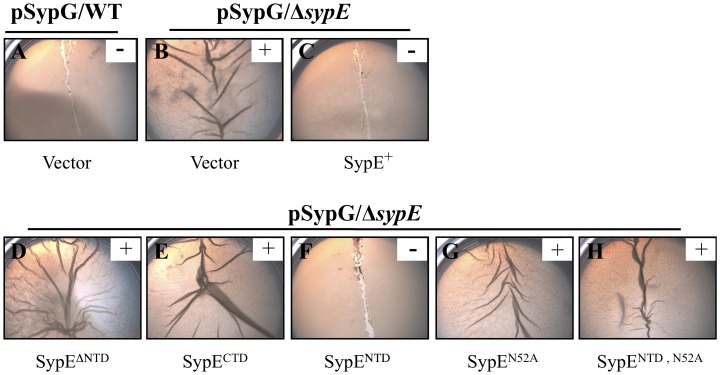
Inhibition of SypG-induced pellicle formation by SypE. The *sypG* expression plasmid (pCLD56) was introduced into either wild-type cells [A] or Δ*sypE* mutant cells [KV3299] carrying either empty vector (pVSV105) [B] or the indicated SypE-complementation plasmids: full-length SypE (pCLD48)[C], SypE^ΔNTD^ (pARM3)[D], SypE^CTD^ (pCLD67)[E], SypE^NTD^ (pCLD64)[F], SypE^N52A^ (pARM4)[G], SypE^NTD, N52A^ (pCLD65)[H]. Strains were cultured statically in LBS medium and pellicle formation was assessed 48 h post-inoculation. A pipette tip was dragged over the surface of the air-liquid interface to visualize the pellicle. (–) denotes a weak, easily disrupted pellicle. (+) denotes a strong, detectable pellicle. Images are representative of at least three independent experiments.

To better understand the mechanism by which SypE inhibits SypG-induced biofilm formation, we utilized a complementation approach. Briefly, we generated a set of plasmids with *sypE* alleles that contained or lacked the individual SypE domains (Fig. 1A). We then co-expressed *sypG* with the various *sypE* alleles in the Δ*sypE* mutant and assessed the resulting biofilm phenotypes. As previously reported, wild-type cells failed to produce SypG-induced biofilms, indicated by the smooth colony morphology on solid agar media and little to no pellicle formation in static liquid culture (Fig. 2A and 3A) [12]. In contrast, Δ*sypE* cells exhibited dramatic SypG-induced biofilm phenotypes, including wrinkled colony formation and robust pellicle formation (Figs. 2B and 3B, respectively) [12]. Co-expression of wild-type *sypE* fully complemented the *sypE* mutant and restored inhibition of biofilms, resulting in smooth colony morphology and lack of robust pellicle formation similar to wild-type cells (Fig. 2C and 3C, respectively).

We next examined the regulatory activity of a SypE variant (SypE^ΔNTD^; Fig. 1A) that lacks the N-terminal 135 amino acids, and found that it failed to complement the *sypE* mutant, but instead permitted wrinkled colony (Fig. 2D) and pellicle (Fig. 3D) formation similar to vector-containing cells (Figs. 2B and 3B, respectively). Similarly, expression of the C-terminal domain alone (SypE^CTD^; Fig. 1A) failed to complement the *sypE* mutant and inhibit biofilm formation (Figs. 2E and 3E). These results indicate that inhibition of SypG-induced biofilms requires the N-terminal domain of SypE. We previously reported that both the SypE^ΔNTD^ and *sypE* ^CTD^ alleles were capable of promoting biofilms induced by *rscS* overexpression, indicating the resulting proteins were produced and functional [19]. Western blot analyses also confirmed that the SypE^ΔNTD^ and *sypE* ^CTD^ proteins were indeed stably produced (Fig. 2I). These results indicate that inhibition of SypG-induced biofilms requires the N-terminal domain of SypE.

To further investigate the potential inhibitory role of the N-terminal domain, we expressed SypE^NTD^, which contains only the 140 amino acids of the N-terminal domain of SypE (Fig. 1A). We found that SypE^NTD^ fully complemented the *sypE* mutant, restoring inhibition of both wrinkled colony and pellicle formation (Fig. 2F and 3F, respectively). Together, these results demonstrate that the N-terminal domain of SypE is both necessary and sufficient to inhibit SypG-induced biofilm formation.

### Inhibitory Activity of the N-terminal Domain Requires Conserved Asparagine Residue N52A

The N-terminal domain of SypE exhibits sequence similarity to HPK (histidine protein kinase)-like serine kinases [18]. Importantly, SypE contains a conserved asparagine residue (N52) [18], which in HPK-like serine kinases participates in Mg^2+^ ion coordination and the binding of ATP to the nucleotide pocket [29]. In several characterized HPK-like serine kinases, mutagenesis of this asparagine residue results in the loss of serine kinase activity [30], [31]. Similarly, we found that this conserved asparagine was required for a mutant SypE variant to inhibit biofilms induced by RscS [19]. To ask whether the ability of wild-type SypE to inhibit SypG-induced biofilms also required this conserved asparagine, we assessed the inhibitory activity of a SypE mutant carrying an alanine substitution at this site (SypE^N52A^). We found that expression of SypE^N52A^ failed to complement the Δ*sypE* mutant: the colonies exhibited strong SypG-induced wrinkling and pellicle formation (Figs. 2G and 3G). Similarly, this mutation in the context of the inhibitory N-terminal domain alone (SypE^NTD, N52A^) resulted in the complete loss of inhibitory activity: cells co-expressing *sypE* ^NTD, N52A^ and *sypG* exhibited both wrinkled colony morphology and pellicle formation (Figs. 2H and 3H, respectively). The failure to complement cannot be attributed to protein instability, as epitope-tagged versions of both the SypE^N52A^ and SypE^NTD, N52A^ proteins are stably expressed [19]. From these results, we conclude that the inhibitory activity of SypE’s N-terminal, kinase domain requires conserved residue N52, consistent with that seen for other characterized HPK-like serine kinases.

### Co-overexpression of SypA and SypG Permits Biofilm Formation

**Figure 4:**
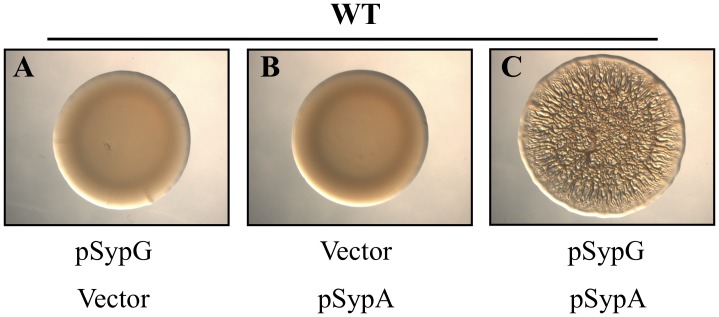
Co-overexpression of *sypG* and *sypA* permits biofilm formation. Biofilm formation by wild-type *V. fischeri* cells carrying either the *sypG* overexpression plasmid (pARM9) [A], the *sypA* overexpression plasmid (pARM13) [B], or both [C]. For A and B, the indicated vectors are pKV282 and pVSV105, respectively. The strains were cultured in LBS broth containing Tet and Cm. Cultures were spotted onto agar plates and wrinkled colony morphology was assessed at 48 h post-spotting. Images are representative of at least three independent experiments.

Our results indicate that SypE inhibits SypG-induced biofilms through the activity of its N-terminal, serine kinase domain. These findings suggest that SypE primarily functions as a kinase under SypG-inducing conditions, and likely inhibits biofilms by phosphorylating a downstream target protein. We hypothesized that SypE exerts negative control over SypG-induced biofilms by phosphorylating the *syp*-encoded regulator SypA, which we recently identified as a downstream target of SypE (Fig. 1B) [20]. SypA, a putative anti-sigma factor antagonist, is required for biofilm formation, and phosphorylation of SypA inhibits its activity [20]. We reasoned that if SypE prevents SypG-induced biofilms through phosphorylation of SypA, then co-overexpression of *sypG* and *sypA* may result in sufficiently high levels of SypA such that some of it could escape phosphorylation and thus inhibition by SypE. To test this, we co-overexpressed *sypA* and *sypG* from compatible plasmids in a wild-type (*sypE* ^+^) background and assessed biofilm formation. As controls, we first evaluated strains overexpressing either *sypG* or *sypA* alone, and found that biofilm formation was not induced (Fig. 4A and 4B, respectively). In contrast, cells co-overexpressing both *sypG* and *sypA* exhibited robust biofilm formation (Fig. 4C). These data indicate that providing excess SypA overcomes the inhibition of SypG-induced biofilms by SypE, and further suggest that SypA functions downstream of SypE and is likely the target for SypE’s inhibitory, kinase activity. In addition, the fact that biofilms were only induced upon co-overexpression of *sypG* and *sypA*, but not when *sypA* was overexpressed alone, indicates that *sypG* is still required to induce *syp* transcription and the production of the Syp structural proteins (Fig. 1B).

### Phosphorylation of SypA is Required for Inhibition of SypG Biofilms by SypE

**Figure 5:**
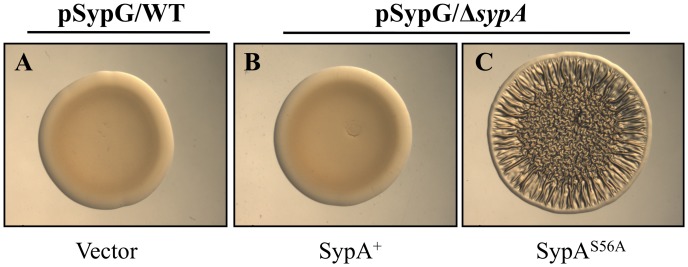
A *sypA* ^S56A^ mutant permits SypG-induced biofilm formation. Assessment of SypG-induced wrinkled colony formation by *sypG*-overexpressing (pCLD56) wild-type cells [A], and *sypG*-overexpressing Δ*sypA* cells complemented with either wild-type *sypA* ^+^ (KV5479) [B] or the *sypA* ^S56A^ allele (KV5481) [C]. Cultures were spotted onto LBS medium containing Cm at 28°C and wrinkled colony formation was assessed at 48 h post spotting. Images are representative of at least three independent experiments.

Our data predicted that the inhibition of biofilm formation by SypE depends upon its ability to phosphorylate SypA. If so, then it should be possible to overcome SypE’s inhibition of biofilm formation with a mutant of SypA that does not become phosphorylated. We thus assayed SypG-induced biofilm formation by cells that expressed the *sypA* ^S56A^ allele in single copy in the chromosome; the resulting mutant protein contains a substitution at a conserved serine required for phosphorylation, and fails to become phosphorylated either *in vivo* when *rscS* is overexpressed or *in vitro* [20]. We found that *sypE* ^+^ cells expressing SypA^S56A^, but not those that expressed wild-type SypA or contained the empty cassette, formed robust biofilms upon overexpression of *sypG* (Fig. 5). These data demonstrate that the non-phosphorylatable SypA^S56A^ mutant is insensitive to the inhibitory activity of SypE, thus supporting the hypothesis that SypE inhibits the formation of SypG-induced biofilms via phosphorylation of SypA.

### SypE Promotes Phosphorylation of SypA in *sypG*-overexpressing Cells

**Figure 6:**
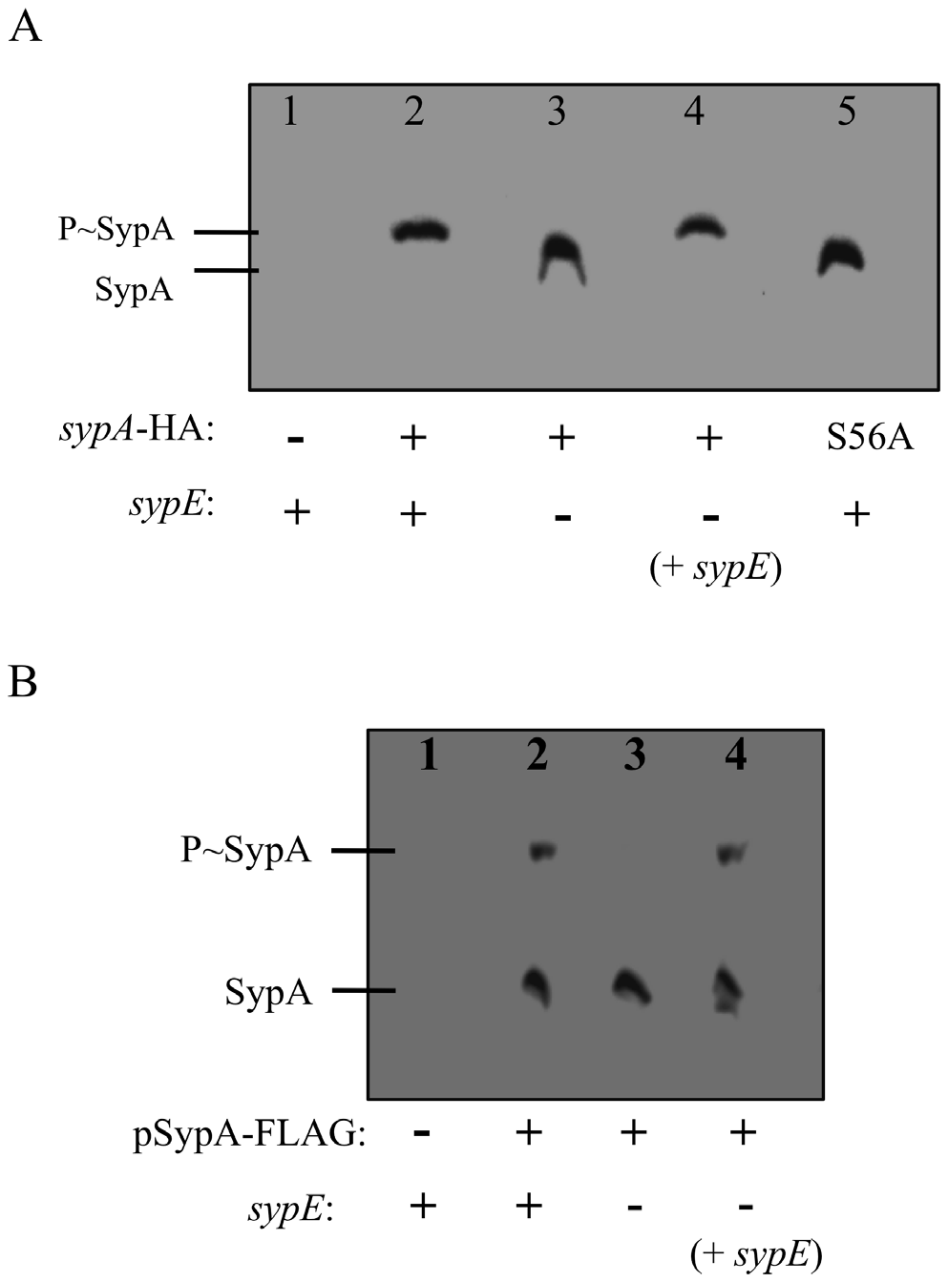
Assessment of SypA phosphorylation *in vivo.* Soluble lysates from the indicated *V. fischeri* strains were resolved by SDS-PAGE on 30 µM Phos-tag™ acrylamide gels and the proteins were detected by western blot analysis using anti-HA antibody (A) or anti-FLAG antibody (B). (A) Phos-tag™ analysis of soluble cell lysates from *V. fischeri* strains expressing HA-tagged *sypA* in single-copy. SypG-expressing (pCLD56) Δ*sypA* cells containing untagged *sypA* [KV5479] [lane 1] or HA-tagged wild-type *sypA* [KV6578] [lane 2]; Δ*sypA* Δ*sypE* cells expressing HA-tagged wild-type *sypA* ^+^ [KV6580] and carrying pCLD56[lane 3]; Δ*sypA ΔsypE* expressing HA-tagged wild-type *sypA* ^+^ [KV6580] and carrying pCLD56 and pSypE plasmid (pCLD48)[lane 4]; Δ*sypA* cells expressing HA-tagged *sypA* ^S56A^ [KV6579] and carrying pCLD56 [lane 5]. (+) indicates cells expressing wild-type *sypA*-HA and/or *sypE*. (S56A) indicates cells expressing *sypA* ^S56A^. (–) indicates cells expressing untagged *sypA* or deleted for *sypE*. (B) Phos-tag™ analysis of soluble cell lysates from *sypA-* and *sypG-*overexpressing *V. fischeri* strains. Δ*sypA* cells [KV4715] carrying the SypG plasmid (pARM9) and either untagged SypA plasmid (pARM13) [lane 1] or FLAG-tagged SypA plasmid (pARM35) [lane 2]; Δ*sypA* Δ*sypE* cells [KV4716] carrying pARM9 and pARM35 [lane 3]; Δ*sypA* Δ*sypE* cells complemented with wild-type *sypE* [KV5649] carrying pARM9 and pARM35 [lane 4]. (–) indicates cells expressing untagged *sypA* or deleted for *sypE*. Images are representative of at least three independent experiments.

Our genetic data to date suggested that, in cells that overexpress *sypG*, SypE functions as a kinase to phosphorylate SypA. To assess this prediction directly, we evaluated the *in vivo* phosphorylation state of SypA under both biofilm-inhibitory conditions (i.e. *sypG*-overexpressing wild-type cells) and biofilm-permissive conditions (i.e. *sypG*-overexpressing Δs*ypE* cells). Briefly, we introduced an epitope-tagged wild-type *sypA* allele (containing a C-terminal HA tag) in single copy in the chromosome (at the Tn*7* site) of either Δ*sypA* cells or Δ*sypA* Δ*sypE* cells. We then overexpressed *sypG* and assessed the *in vivo* phosphorylation state of SypA by resolving cell lysates on SDS-PAGE gels containing Phos-tag™ acrylamide. This reagent permits the separation of phosphorylated and non-phosphorylated forms of proteins by preferentially binding to and retarding the migration of phosphorylated proteins [32], [33]. SypA-HA proteins were detected by western blot analysis using an anti-HA antibody. As observed in figure 6A, *sypG*-overexpressing wild-type cells (lane 2) exhibited a single, upper band corresponding to phosphorylated SypA (SypA∼P). These data indicate that in wild-type cells overexpressing *sypG*, the majority of, if not all, SypA protein is in the phosphorylated state. In contrast, *sypG*-overexpressing Δ*sypE* cells (which are competent to produce biofilms) exhibited a single, lower band representing unphosphorylated SypA (Fig. 6A, lane 3). Complementation with a wild-type allele of *sypE* (SypE^+^) restored SypA phosphorylation as indicated by the presence of the shifted SypA band (SypA∼P) (Fig. 6A, lane 4). Finally, we confirmed that the SypA^S56A^ mutant fails to become phosphorylated in *sypG*-overexpressing cells (Fig. 6A, lane 5). These results demonstrate that SypE promotes SypA phosphorylation under SypG-inducing conditions, and corroborate our biofilm assays indicating that, under SypG-inducing conditions, SypE functions as a kinase. Importantly, they verify that phosphorylation of SypA is critical for biofilm inhibition.

To further test the hypothesis that biofilm formation requires unphosphorylated SypA, we examined the phosphorylation state of SypA in cells co-overexpressing *sypG* and *sypA*. We previously found that the co-overexpression of *sypG* and *sypA* promotes biofilm formation in wild-type (*sypE* ^+^) cells (Fig. 4). We hypothesized that the overexpression of *sypA* results in an excess of SypA protein, such that a pool of SypA escapes phosphorylation by SypE and is thus active to promote SypG-induced biofilms. We found that wild-type cells expressing *sypA* and *sypG* consistently exhibited two bands: a predominant, lower band corresponding to unphosphorylated SypA and a faint, upper band corresponding to phosphorylated SypA (Fig. 6B, lane 2). These data thus demonstrate that overexpression of *sypA* indeed provides an excess pool of unphosphorylated SypA protein. In agreement with our previous Phos-tag™ experiments, SypA phosphorylation depended upon the presence of SypE, as cells deleted for *sypE* exhibited only the lower SypA band corresponding to non-phosphorylated SypA (Fig. 6B, lane 3). Finally, complementation with *sypE* caused a subset of SypA to become phosphorylated under these conditions (Fig. 6B, lane 4). Together, these results support our earlier Phos-tag™ studies indicating that biofilm formation requires unphosphorylated, active SypA.

### SypE Regulates SypG-induced Biofilm Formation Downstream of *syp* Activation

Although our data indicate that SypE-mediated phosphorylation of SypA inhibits biofilm formation upon *sypG* overexpression, it does not reveal the level at which SypE (via SypA) exerts its control. SypG regulates biofilm formation by inducing transcription of the *syp* locus, which is required for both biofilm formation in laboratory culture and the formation of bacterial aggregates on the surface of the squid light organ during colonization [5], [12]. Therefore, one formal possibility is that SypE impacts transcription of the *syp* locus through SypA. To determine if SypE affects *syp* transcription, we utilized a *sypA* promoter-*lacZ* fusion inserted in single copy in the chromosome of the wild-type and Δ*sypE* strains. We then assessed *sypA* promoter activity by measuring β-galactosidase activity upon co-expression of *sypG* and *sypE*.

**Figure 7:**
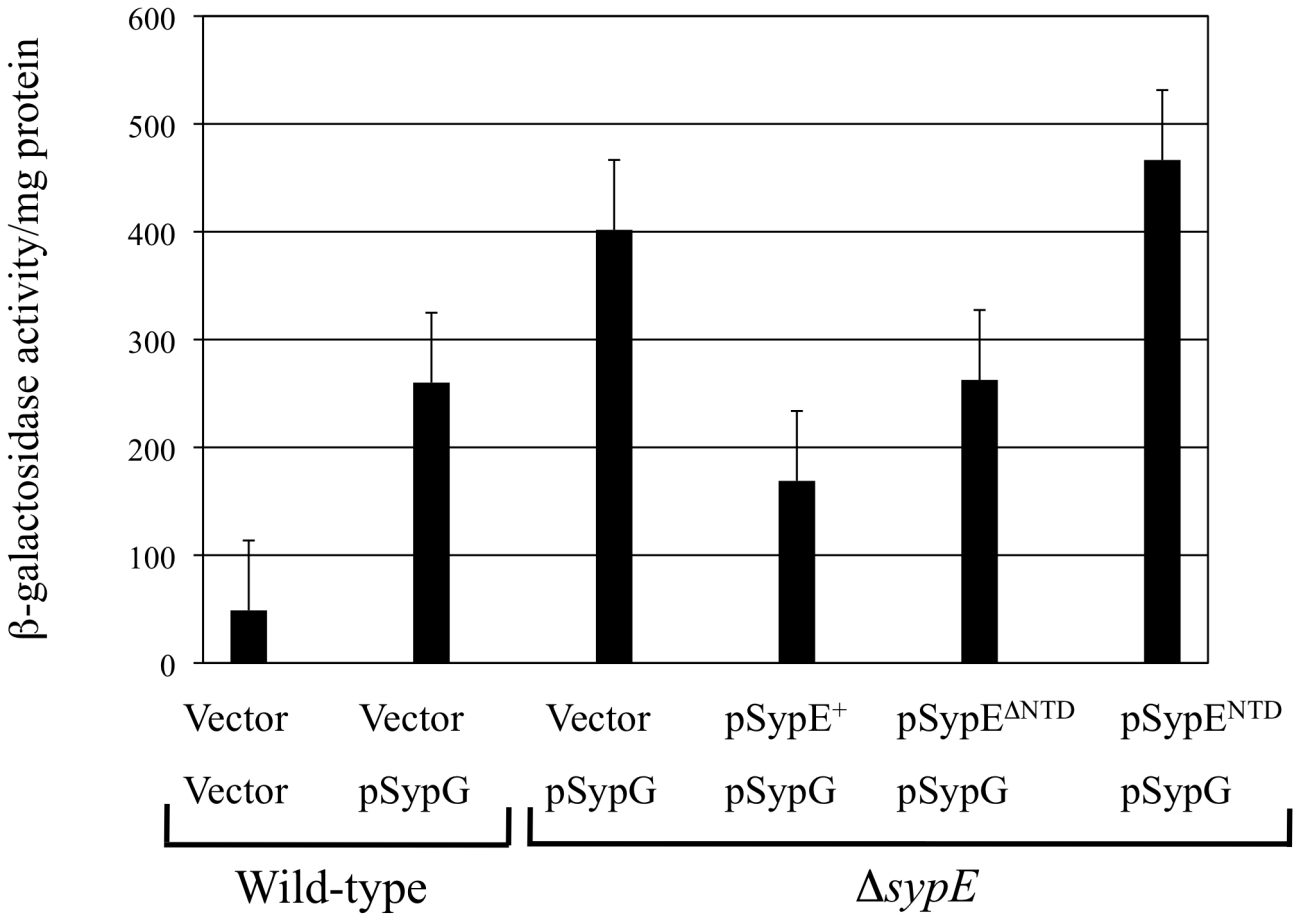
Impact of SypE on *syp* locus activation. Transcription of the *syp* locus was monitored using a β-galactosidase activity assay. A transcriptional reporter construct consisting of the *sypA* promoter region fused upstream of a promoterless *lacZ* gene was inserted at the chromosomal Tn*7* site of wild-type [KV3246] or Δ*sypE* [KV4926] cells carrying the pSypG plasmid (pCLD56) and indicated SypE expression plasmids: wild-type SypE (pCLD48), SypE^ΔNTD^ (pARM3), and SypE^NTD^ (pCLD64). Vector corresponds to pVSV105 or, in the case of wild-type carrying two vectors, pVSV105 and pKV282. Cells were grown in LBS containing Tc and Cm for 24 h. Results shown are representative of at least 3 independent experiments. Error bars indicated the standard deviation.

As previously observed [12], wild-type cells overexpressing *sypG* exhibited high levels of *syp* transcription, as indicated by the significant increase in β-galactosidase activity relative to vector control cells (Fig. 7). Compared to the wild type, cells deleted for *sypE* exhibited a moderate (∼1.5-fold), but consistent, increase in SypG-induced *syp* transcription (Fig. 7). Co-expression of wild-type *sypE* and *sypG* in the Δ*sypE* mutant restored *syp* transcription to near wild-type levels (Fig. 7). These results suggest that SypE does have a minor effect on SypG-induced *syp* transcription.

To further determine whether SypE indeed controls biofilm formation at the level of *syp* activation, we asked whether inhibition of SypG-induced transcription required the SypE N-terminal serine kinase domain. We first tested whether expression of SypE^ΔNTD^, a *sypE* mutant that lacks the N-terminal domain and thus its ability to inhibit biofilm formation, impacted activation of *syp* transcription by SypG. Surprisingly, we found that co-expression of SypE^ΔNTD^ and *sypG* in Δ*sypE* cells resulted in a decrease in *syp* transcription similar to cells expressing wild-type *sypE*, a result opposite to the observed biofilm phenotype (Fig. 7). These results suggested that while SypE may have a slight impact on *syp* transcription, it is not sufficient to account for the observed effects of SypE on SypG-induced biofilm formation. In support of this, we found that expression of the N-terminal domain alone (SypE^NTD^), which is sufficient to inhibit SypG-induced biofilm formation, had no observable impact on *syp* transcription (Fig. 7; compare with Δ*sypE* vector control). From these data, we conclude that the minor effect of SypE on SypG-induced *syp* transcription is insufficient to account for the dramatic effects on biofilm formation, and thus SypE must exert its regulatory effect downstream of *syp* transcriptional activation.

### Deletion of *sypE* Confers a Competitive Colonization Advantage

**Figure 8:**
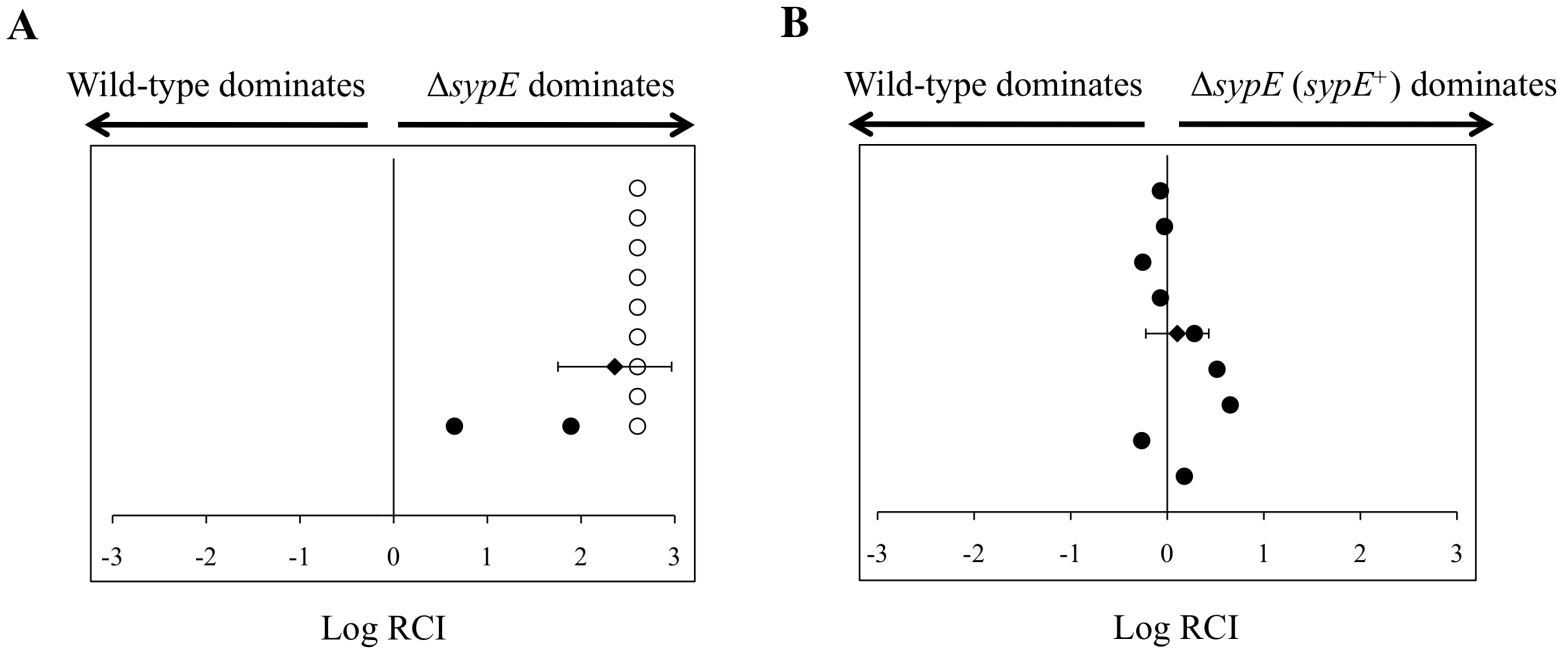
Deletion of *sypE* promotes host colonization. Competitive colonization assay with *sypG*-overexpressing wild-type (WT) and *sypE* mutant strains. Newly hatched squid were exposed to a mixed inoculum of WT carrying the pSypG plasmid (pCLD56) and either Δ*sypE* cells [KV4390] (A) or Δ*sypE* cells complemented with wild-type *sypE* ^+^ [KV4819] (B) and carrying pCLD56. The Log RCI is plotted on the x-axis. The position of the circles on the y-axis is merely for spacing. Each circle represents a single animal. Open symbols indicate animals containing no WT cells. The black diamond and error bars indicate the average Log RCI and standard deviation for the indicated data set. Data shown are representative of at least three independent experiments.

The ability of *V. fischeri* to produce *syp*-dependent biofilms in laboratory culture directly correlates with the ability of the bacteria to efficiently colonize host juvenile squid [11], [14], [19]. However, we previously observed that the deletion of *sypE* in otherwise wild-type cells did not significantly impact host colonization [19], [34]. We therefore questioned whether the presence of *sypE* would impact colonization when *sypG* is overexpressed. In other words, would the ability of the *sypG*-overexpressing *sypE* mutant cells to produce robust biofilms also promote colonization competence, or would the absence of *sypE* have no impact? To assess this question, we examined the ability of *sypG*-overexpressing wild-type and Δ*sypE* strains to competitively colonize juvenile squid. Newly hatched juvenile squid were inoculated with a 1∶1 mixture of the two strains, one of which carried an erythromycin resistance (Em^R^) cassette on the chromosome. Upon colonization, the squid were homogenized and the relative amounts of the individual strains were determined by calculating the percentage of Em^R^ and Em^S^ colonies. As shown in Figure 8A, Δ*sypE* cells dramatically outcompeted wild-type cells for host colonization. These data suggest that upon overexpression of *sypG*, deletion of *sypE* confers a competitive colonization advantage. To determine whether the loss of *sypE* was responsible for the colonization advantage observed in the Δ*sypE* strain, we complemented the Δ*sypE* strain with a wild-type allele of *sypE in trans* in the chromosome. Indeed, the SypE-complemented (SypE^+^) cells failed to outcompete wild-type cells, when both were overexpressing *sypG* (Fig. 8B). These results reveal a critical role for SypE in inhibiting both biofilm formation and host colonization. Furthermore, they provide additional correlations between *syp*-dependent biofilm formation in culture and the ability to promote host colonization *in vivo.*

## Discussion

In this study, we investigated the role of the SypE in the regulation of SypG-dependent biofilm formation and host colonization in *V. fischeri*. Our interest in this area was prompted by an earlier report demonstrating that SypE permitted RscS-induced biofilms, yet inhibited biofilms inhibited by *sypG* overexpression [12]. Thus, SypE appeared to function in distinct ways under the two conditions. SypE is an unusual response regulator with both serine kinase and serine phosphatase activities [18], [19], [20]. To better understand the role of SypE as a negative regulator of *syp*-dependent biofilm formation, and to determine the mechanism behind the distinct phenotypes displayed by the *rscS* and *sypG* overexpression strains, we assessed SypE function under *sypG*-overexpressing conditions.

First, we performed a structure-function analysis to probe the regulatory activities of this novel response regulator. Through complementation studies, we observed that the N-terminal serine kinase domain is both required and sufficient to inhibit SypG-induced biofilm formation. The inhibitory activity of the isolated N-terminal domain (SypE^NTD^) absolutely required residue N52, a conserved asparagine that is necessary for ATP binding and kinase activity in other characterized serine kinases [29], [30]. These results parallel those observed for the role of SypE in RscS-induced biofilm formation: expression of SypE^NTD^ alone also inhibited biofilm formation in a manner that depended on N52 [19]. Future efforts are required to determine what other residues contribute to SypE-mediated inhibition of biofilms. Unlike our results with the N-terminal domain, however, our results with respect to the C-terminal domain differed under *rscS* and *sypG*-overexpressing conditions: whereas the C-terminal domain promoted RscS-induced biofilms [19], we failed to observe any impact of this domain in promoting SypG-induced biofilm formation (Fig. 2 and data not shown). It remains possible that the C-terminal serine phosphatase domain does promote SypG-induced biofilms, but that this impact is not readily apparent under the current experimental conditions. Indeed, SypG-mediated induction of *syp* transcription is stronger than that induced by RscS, and correspondingly, biofilm formation occurs at an accelerated pace by *sypG*-overexpressing Δ*sypE* strains relative to *rscS*-overexpressing strains (Morris and Visick, unpublished data). Thus, it is more difficult to assess a subtle increase in biofilm formation under the SypG conditions.

Next, we used both genetic and biochemical approaches to assess whether SypE inhibited SypG-induced biofilms by promoting the phosphorylation, and thus inactivation, of SypA, a critical regulator required for biofilm development. Co-overexpression of *sypG* and *sypA* in *sypE* ^+^ cells promoted biofilm formation, indicating that high levels of SypA can overcome the inhibitory activity of SypE, presumably because some SypA escapes phosphorylation (Fig. 1B and 6B). In further support of this conclusion, expression from the chromosome of a *sypA* mutant that does not become phosphorylated (SypA^S56A^) also permitted the formation of SypG-induced biofilms in wild-type (SypE^+^) cells. Finally, we found that SypA was predominantly phosphorylated under biofilm-inhibiting conditions (i.e., upon overexpression of *sypG* in wild-type cells), but unphosphorylated under biofilm-permissive conditions (i.e. upon overexpression of *sypG* in Δ*sypE* cells). The ability to evaluate the phosphorylation state of SypA *in vivo* provides a critical link between the biochemical proof of activity and the genetic and phenotypic analyses. Importantly, these findings demonstrate that SypE inhibits SypG-induced biofilm formation by functioning as a kinase to phosphorylate SypA (Fig. 1B). They also corroborate our previous study demonstrating that SypA is largely unphosphorylated under biofilm-permissive conditions, specifically in *rscS*-overexpressing wild-type cells [20].

Studies are underway to determine the mechanism by which the SypE-SypA pathway controls biofilm formation. Here, we asked whether SypE (via SypA) exerts any impact on biofilm formation through affecting *syp* transcription. We found that the deletion and/or overexpression of full-length *sypE* or a subset of mutant *sypE* alleles resulted in a slight, yet reproducible, impact on *syp* transcription. However, the observed effects on transcription were not sufficient to account for the observed biofilm phenotypes. For example, we found that a SypE mutant lacking the N-terminal domain (SypE^ΔNTD^) and, thus, lacking inhibitory activity retained the ability to decrease SypG-induced transcription. We hypothesize that SypE’s REC domain may be responsible for the observed impact on *syp* transcription. SypE, like SypG, appears to function below RscS in the regulatory cascade [12], [19]. If these two response regulators do indeed receive signals from the same upstream sensor kinase, then the REC domains of SypE and SypG may compete for phosphorylation, resulting in a decrease in SypG activation and *syp* transcription. In support of this possibility, expression of SypE^NTD^, a SypE derivative that lacks the REC domain but retains biofilm-inhibitory activity, had no observable impact on *syp* transcription. Due to the lack of correlation between biofilm formation and the minor impacts of a subset of *sypE* alleles on *syp* transcription, we conclude that SypE functions at a level below *syp* transcription. In support of this conclusion, our preliminary studies indicate that expression of *sypA*, although critical for biofilm formation, exerts no impact *syp* transcription (Morris and Visick, unpublished data).

Finally, we found that the ability of *V. fischeri* to form biofilms in culture directly correlates with colonization efficiency *in vivo*, a result that is consistent with previous studies [11], [14], [19]. In particular, we observed that the enhanced ability of the Δ*sypE* strain to form biofilms upon overexpression of *sypG* provides these cells with a competitive colonization advantage, presumably due to enhanced aggregation outside of the symbiotic light organ. We found that this colonization advantage, similar to biofilm formation, depended upon loss of *sypE* as complementation with *sypE in trans* in the chromosome abolished the colonization advantage. Since previous studies had found no substantial colonization phenotype upon the deletion of *sypE* [19], [34], why was SypE so critical here? Our results to date indicate that wild-type cells inactivate SypE during the early stages of squid colonization, and thus its loss would have relatively little impact on colonization initiation [19]. We speculate that the levels of SypE produced under *sypG*-overexpression conditions are greater than can be inactivated through the mechanisms in place. In support of this idea, we have found that SypE expressed from a multi-copy plasmid inhibits biofilm formation, even under *rscS*-overexpression conditions (Morris and Visick, unpublished data). In any event, the significant inhibitory effect of SypE on colonization under SypG-inducing conditions underscores the critical importance of biofilm formation in promoting symbiotic host colonization.

This study also emphasizes the strict control *V. fischeri* exerts over biofilm formation (Fig. 1B). The finding that SypE inhibits biofilms under SypG-inducing conditions, yet permits biofilms upon overexpression of *rscS*, suggests that SypE could function to prevent aberrant biofilm formation induced by the phosphorylation, and thus activation, of SypG in the absence of RscS signaling/activation. It is currently unclear under what conditions SypG may become phosphorylated, and induce *syp* transcription, without signaling through RscS. It remains possible that SypG may receive phosphoryl groups and become activated through other regulatory inputs in addition to RscS. Recently, Ray and Visick (2012) demonstrated that regulatory components within the Lux phosphorelay controlling bioluminescence also impact the regulation of *syp* biofilm formation. Importantly, this impact on *syp* biofilms appears to occur at the level of SypG and *syp* locus activation [35]. Thus, it is possible that, under conditions in which the Lux phosphorelay promotes SypG activation and *syp* induction, SypE functions as a negative regulator to inactivate SypA and prevent biofilm formation. Alternatively, SypE may be important for preventing biofilm formation under conditions in which SypG might get phosphorylated due to inadvertent “cross-talk” from other sensor kinases that are activated. These possibilities remain to be investigated.

In summary, this work provides further insight into the mechanism by which the regulator SypE restricts biofilm formation and host colonization by *V. fischeri*. Furthermore, it emphasizes the critical role of SypE in regulating biofilm formation, particularly when RscS is not activating the pathway. Together, our data suggest that SypE may function to restrict biofilms to conditions in which RscS becomes activated (i.e. upon interaction with host squid), and thus to coordinate *syp* biofilm production with host colonization.

## References

[1] Hall-Stoodley L, Costerton JW, Stoodley P (2004) Bacterial biofilms: from the natural environment to infectious diseases. Nat Rev Microbiol 2: 95–108. 15040259 10.1038/nrmicro821

[2] Hogan D, Kolter R (2002) Why are bacteria refractory to antimicrobials? Curr Opin Microbiol 5: 472–477. 12354553 10.1016/s1369-5274(02)00357-0

[3] Karatan E, Watnick P (2009) Signals, regulatory networks, and materials that build and break bacterial biofilms. Microbiol Mol Biol Rev 73: 310–347. 19487730 10.1128/MMBR.00041-08 PMC2698413

[4] Kuchma SL, O’Toole GA (2000) Surface-induced and biofilm-induced changes in gene expression. Curr Opin Biotechnol 11: 429–433. 11024358 10.1016/s0958-1669(00)00123-3

[5] Yip ES, Grublesky BT, Hussa EA, Visick KL (2005) A novel, conserved cluster of genes promotes symbiotic colonization and sigma-dependent biofilm formation by *Vibrio fischeri* . Mol Microbiol 57: 1485–1498. 16102015 10.1111/j.1365-2958.2005.04784.x

[6] Croxatto A, Lauritz J, Chen C, Milton DL (2007) *Vibrio anguillarum* colonization of rainbow trout integument requires a DNA locus involved in exopolysaccharide transport and biosynthesis. Environ Microbiol 9: 370–382. 17222135 10.1111/j.1462-2920.2006.01147.x

[7] Ruby EG (2008) Symbiotic conversations are revealed under genetic interrogation. Nat Rev Microbiol 6: 752–762. 18794913 10.1038/nrmicro1958 PMC3579588

[8] Visick KL, Ruby EG (2006) *Vibrio fischeri* and its host: it takes two to tango. Curr Opin Microbiol 9: 632–638. 17049299 10.1016/j.mib.2006.10.001

[9] Nyholm SV, McFall-Ngai MJ (2004) The winnowing: establishing the squid-vibrio symbiosis. Nat Rev Microbiol 2: 632–642. 15263898 10.1038/nrmicro957

[10] Nyholm SV, Stabb EV, Ruby EG, McFall-Ngai MJ (2000) Establishment of an animal-bacterial association: recruiting symbiotic *vibrios* from the environment. Proc Natl Acad Sci U S A 97: 10231–10235. 10963683 10.1073/pnas.97.18.10231 PMC27829

[11] Yip ES, Geszvain K, DeLoney-Marino CR, Visick KL (2006) The symbiosis regulator *rscS* controls the syp gene locus, biofilm formation and symbiotic aggregation by *Vibrio fischeri* . Mol Microbiol 62: 1586–1600. 17087775 10.1111/j.1365-2958.2006.05475.x PMC1852533

[12] Hussa EA, Darnell CL, Visick KL (2008) RscS functions upstream of SypG to control the syp locus and biofilm formation in *Vibrio fischeri* . J Bacteriol 190: 4576–4583. 18441059 10.1128/JB.00130-08 PMC2446822

[13] Visick KL, Skoufos LM (2001) Two-component sensor required for normal symbiotic colonization of *Euprymna scolopes* by *Vibrio fischeri* . J Bacteriol 183: 835–842. 11208780 10.1128/JB.183.3.835-842.2001 PMC94949

[14] Shibata S, Yip ES, Quirke KP, Ondrey JM, Visick KL (2012) Roles of the structural symbiosis polysaccharide (syp) genes in host colonization, biofilm formation and polysaccharide biosynthesis in Vibrio fischeri. J Bacteriol. 10.1128/JB.00707-12 PMC3510638 23042998

[15] Beyhan S, Tischler AD, Camilli A, Yildiz FH (2006) Transcriptome and phenotypic responses of *Vibrio cholerae* to increased cyclic di-GMP level. J Bacteriol 188: 3600–3613. 16672614 10.1128/JB.188.10.3600-3613.2006 PMC1482859

[16] Shikuma NJ, Fong JC, Odell LS, Perchuk BS, Laub MT, et al (2009) Overexpression of VpsS, a hybrid sensor kinase, enhances biofilm formation in *Vibrio cholerae.* . J Bacteriol 191: 5147–5158. 19525342 10.1128/JB.00401-09 PMC2725581

[17] Simm R, Morr M, Kader A, Nimtz M, Romling U (2004) GGDEF and EAL domains inversely regulate cyclic di-GMP levels and transition from sessility to motility. Mol Microbiol 53: 1123–1134. 15306016 10.1111/j.1365-2958.2004.04206.x

[18] Morris AR, Visick KL (2010) Control of biofilm formation and colonization in *Vibrio fischeri*: a role for partner switching? Environ Microbiol 12: 2051–2059. 21966901 10.1111/j.1462-2920.2010.02269.x PMC2988913

[19] Morris AR, Darnell CL, Visick KL (2011) Inactivation of a novel response regulator is necessary for biofilm formation and host colonization by *Vibrio fischeri* . Mol Microbiol 82: 114–130. 21854462 10.1111/j.1365-2958.2011.07800.x PMC3222273

[20] Morris AR, Visick KL (2013) The response regulator SypE controls biofilm formation and colonization through phosphorylation of the *syp*-encoded regulator SypA in *Vibrio fischeri* . Mol Microbiol 87: 509–525. 23171087 10.1111/mmi.12109 PMC3556205

[21] Boettcher KJ, Ruby EG (1990) Depressed light emission by symbiotic *Vibrio fischeri* of the sepiolid squid *Euprymna scolopes* . J Bacteriol 172: 3701–3706. 2163384 10.1128/jb.172.7.3701-3706.1990 PMC213346

[22] McCann J, Stabb EV, Millikan DS, Ruby EG (2003) Population dynamics of *Vibrio fischeri* during infection of *Euprymna scolopes* . Appl Environ Microbiol 69: 5928–5934. 14532046 10.1128/AEM.69.10.5928-5934.2003 PMC201191

[23] Dunn AK, Millikan DS, Adin DM, Bose JL, Stabb EV (2006) New rfp- and pES213-derived tools for analyzing symbiotic *Vibrio fischeri* reveal patterns of infection and lux expression in situ. Appl Environ Microbiol 72: 802–810. 16391121 10.1128/AEM.72.1.802-810.2006 PMC1352280

[24] Graf J, Dunlap PV, Ruby EG (1994) Effect of transposon-induced motility mutations on colonization of the host light organ by *Vibrio fischeri* . J Bacteriol 176: 6986–6991. 7961462 10.1128/jb.176.22.6986-6991.1994 PMC197071

[25] Davis RW, Botstein D, Roth JR (1980) Advanced bacterial genetics.

[26] Ray VA, Morris AR, Visick KL (2012) A semi-quantitative approach to assess biofilm formation using wrinkled colony development. J Vis Exp: e4035. 10.3791/4035 PMC3466081 22710417

[27] Miller JH (1972) Experiments in molecular genetics. Cold Spring Harbor Laboratory, New York, NY.

[28] Lowry OH, Rosebrough NJ, Farr AL, Randall RJ (1951) Protein measurement with the Folin phenol reagent. J Biol Chem 193: 265–275. 14907713

[29] Dutta R, Inouye M (2000) GHKL, an emergent ATPase/kinase superfamily. Trends Biochem Sci 25: 24–28. 10637609 10.1016/s0968-0004(99)01503-0

[30] Kozak NA, Mattoo S, Foreman-Wykert AK, Whitelegge JP, Miller JF (2005) Interactions between partner switcher orthologs BtrW and BtrV regulate type III secretion in *Bordetella* . J Bacteriol 187: 5665–5676. 16077112 10.1128/JB.187.16.5665-5676.2005 PMC1196064

[31] Yang X, Kang CM, Brody MS, Price CW (1996) Opposing pairs of serine protein kinases and phosphatases transmit signals of environmental stress to activate a bacterial transcription factor. Genes Dev 10: 2265–2275. 8824586 10.1101/gad.10.18.2265

[32] Kinoshita E, Kinoshita-Kikuta E, Takiyama K, Koike T (2006) Phosphate-binding tag, a new tool to visualize phosphorylated proteins. Mol Cell Proteomics 5: 749–757. 16340016 10.1074/mcp.T500024-MCP200

[33] Kinoshita-Kikuta E, Aoki Y, Kinoshita E, Koike T (2007) Label-free kinase profiling using phosphate affinity polyacrylamide gel electrophoresis. Mol Cell Proteomics 6: 356–366. 17088264 10.1074/mcp.T600044-MCP200

[34] Hussa EA, O’Shea TM, Darnell CL, Ruby EG, Visick KL (2007) Two-component response regulators of *Vibrio fischeri*: identification, mutagenesis, and characterization. J Bacteriol 189: 5825–5838. 17586650 10.1128/JB.00242-07 PMC1952042

[35] Ray VA, Visick KL (2012) LuxU connects quorum sensing to biofilm formation in *Vibrio fischeri* . Mol Microbiol 86: 954–970. 23035866 10.1111/mmi.12035 PMC3566283

[36] Stabb EV, Ruby EG (2002) RP4-based plasmids for conjugation between *Escherichia coli* and members of the *Vibrionaceae* . Methods Enzymol 358: 413–426. 12474404 10.1016/s0076-6879(02)58106-4

